# Environmental enrichment affects immunity and reduces disease severity in pigs after co-infection, with stronger effects when applied from birth than from weaning

**DOI:** 10.3389/fvets.2024.1511209

**Published:** 2024-12-09

**Authors:** Brigitte G. C. de Bruijn, Ingrid Danielle Ellen van Dixhoorn, J. Elizabeth Bolhuis, Jan B. W. J. Cornelissen, Norbert Stockhofe-Zurwieden, Marion Kluivers, Johanna M. J. Rebel

**Affiliations:** ^1^Wageningen Livestock Research, Wageningen University & Research, Wageningen, Netherlands; ^2^Adaptation Physiology Group, Wageningen University & Research, Wageningen, Netherlands; ^3^Wageningen Bioveterinary Research, Wageningen University & Research, Lelystad, Netherlands; ^4^Wageningen Environmental Research, Wageningen University & Research, Wageningen, Netherlands

**Keywords:** pigs, enriched housing, early life, PRRSV, *Actinobacillus pleuropneumoniae*, disease susceptibility

## Abstract

We investigated whether environmental enrichment applied at different life stages of pigs affects the susceptibility to and severity of disease by studying immune cell functions around weaning and during nursery, the effects of infection in *ex vivo* models and *in vivo* using a co-infection model of *Porcine Reproductive and Respiratory Syndrome Virus* (PRRSV) followed by an *Actinobacillus pleuropneumoniae* infection. Pigs were either conventionally housed (CCH) or enriched housed throughout life, with enrichment consisting of extra space, rooting materials and co-mingling with another litter before weaning (EEH), or they were switched from conventional to enriched housing at weaning (CEH). Sixty days after birth, ten pigs per treatment were infected with PRRSV followed by an *A. pleuropneumoniae* infection eight days later. Six other pigs per treatment were euthanized before their pen mates were exposed to the co-infection. From these piglets, bronchial-alveolar fluid was collected, and precision cut lung slices were taken to test the effect of the treatments in an *in vitro* infection model. At six days after weaning EEH pigs had higher whole blood cell counts and higher concentrations of IL1ß and TNFα than CCH and CEH pigs. In the *ex vivo* precision cut lung slice model no differences in cytokine response in lung tissue after infection with swine influenza or *A. pleuropneumoniae* were observed between treatments. After experimental co-infection the proportion of EEH pigs with lung lesions (3/10) tended to be lower than in CCH (8/10), with CEH (6/10) being in between. In conclusion, enriched housing from birth reduced disease severity to co-infection with PRRSV and *A. pleuropneumoniae*. Enrichment applied after weaning also seemed to decrease the pathological lung deviations to the co-infection as compared to barren housed pigs, but to a much lower extent.

## 1 Introduction

Infectious diseases are a major health and welfare threat for young pigs in the nursery age. These diseases are often related to persistently present, endemic infections on the farm, which leads to disease in a limited, varying fraction of animals. Diseases in young pigs are caused by bacteria, like *Actinobacillus pleuropneumoniae* or *Streptococcus suis*, viral infections like *Porcine Reproductive and Respiratory Syndrome Virus (PRRSV)* or combinations of these, leading to co-infections, with an aggravated course of disease (1, 2). The resulting clinical diseases as pneumonia or systemic infections cause mortality and morbidity of animals, and also considerable economic losses (3). The susceptibility to develop disease is related to the immunological development in the early phase of life and the imbalance between decreasing passive immunity and building up adaptive immune responses. Immune development is also relying on the innate immune response (4, 5) and orchestrated inflammatory responses after infections. Immune development is therefore critical for adequate responses to infections.

In conventional pig farming, pigs are often reared under stimulus-poor housing conditions. Pigs reared in such conditions often show less activity, play behavior and explorative activities than pigs in enriched housing (6, 7). These behavioral differences may arise from the limitation to express natural behavior (8, 9). Previous studies have shown that environmental enrichment, for instance by supplying materials like straw or peat to meet the behavioral need of pigs to forage and explore, leads to improved health and welfare and affect the immune development. This is indicated by, for instance, fewer days of diarrhea after weaning and less gastric lesions at slaughter in enriched housed pigs compared with pigs reared under conventional housing conditions (10–13), lower display of damaging behaviors like tail biting (14), lower number of skin lesions (15), and a more optimistic affective state (16). Moreover, environmental enrichment has also been shown to increase resilience to stressful events (17), and affect immune function and disease susceptibility (18–21). In line with this, pigs reared in stimulus-poor conditions were shown to differ from those kept in a more enriched environment in immune (re)activity (22–24) and in response to a sickness challenge (17). Moreover, we have demonstrated in an experimental setting that housing conditions also affect the response of pigs to lung disease caused by a low-pathogenic experimental co-infection with porcine reproductive and respiratory virus (PRRSV) and *A. pleuropneumoniae*. Environmental and social enrichment (e.g., rooting substrates like straw and wood shavings, co-mingling of litters, and increased space allowance), which were applied from birth onwards, reduced disease outcome of this lung challenge (21).

Effects of enriched housing on behavior and immune function may depend on in which life stage enrichment is applied. Several studies pinpoint the impact of enrichment in early life, by demonstrating long-lasting effects of pre-weaning environmental enrichment on immune development and behavior, as access to rooting substrates in this life phase reduced nosing and tail biting and improved social behavior after weaning (25–27). It remains uncertain whether enrichment only reduces disease susceptibility when applied from birth onwards, or that introduction of enrichment during later life (e.g., after weaning) is also beneficial. Hence, the aim of this study was to investigate whether the life stage at which enrichment is provided plays a role in immune development and function, response to infection of *ex vivo* lung tissue and *in vivo* disease susceptibility. For the latter, we compared the response to a co-infection model of PRRSV and *A. pleuropneumoniae* in pigs raised in enriched housing from birth onwards to pigs that were housed enriched only after weaning and pigs that were conventionally housed throughout.

## 2 Materials and methods

The established principles of laboratory animal use and the Dutch laws related to animal experiments were adhered to in this study. The Wageningen University Animal Care and Use Committee (Lelystad Department) approved the experiment under number 2017.D-0085.00.

### 2.1 Experimental design, animals, and housing

**Figure 1:**
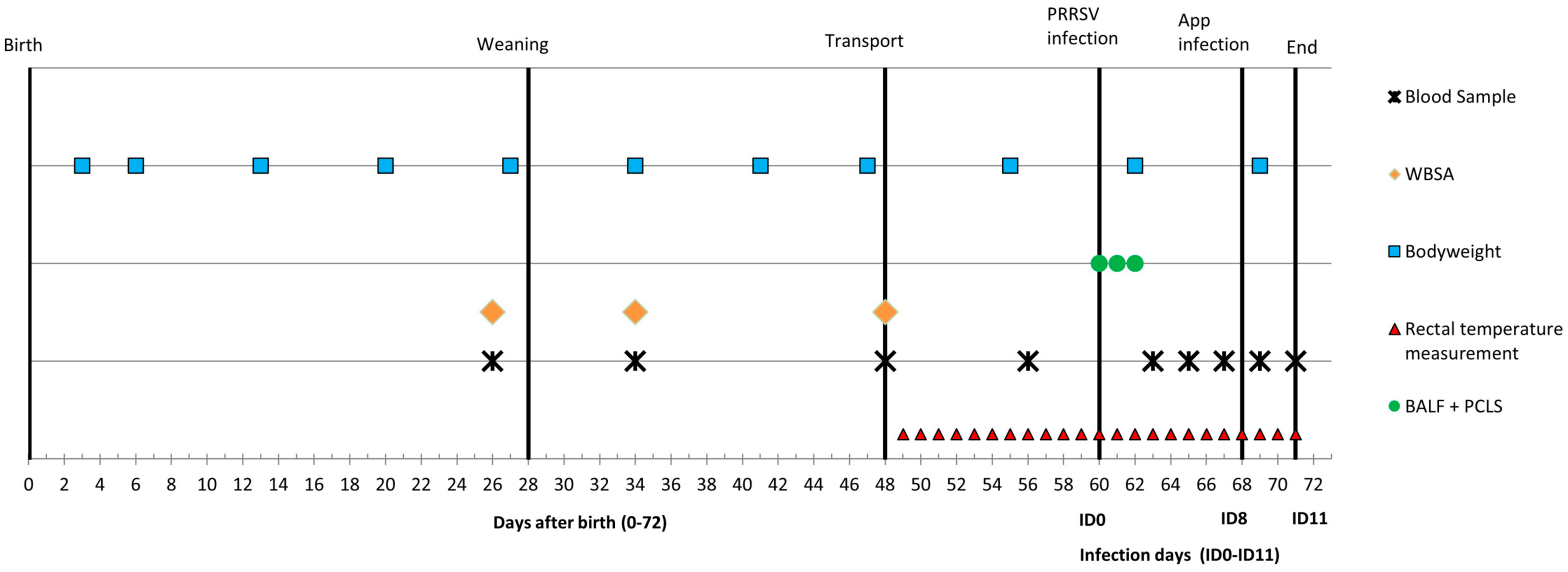
Timeline of the experiment with co-infection of Porcine reproductive and Respiratory Syndrome Virus (PRRSV) and *A. pleuropneumoniae* (APP). WBSA = whole blood stimulation assay, BALF = broncho-alveolar lavage fluid, PCLS = precision-cut lung slices.

For this experiment pigs were selected from the offspring (96 piglets) of eight multiparous Topigs TN70 Z-line sows (range parity: 2–6) housed on a commercial farm with a specific porcine pathogen free (SPF) status. The selected piglets were exposed to one of three treatments: conventional housing before and after weaning (conventional-conventional housing; CCH), enriched housing before and after weaning (enriched-enriched housing; EEH), or conventional housing before and enriched housing after weaning (conventional–enriched housing; CEH). Fourteen pigs per treatment (*n* = 42 in total) were used in an infection challenge (10 infected pigs and four controls per treatment), and from another eight (non-infected) pigs per treatment (*n* = 24 in total) broncho-alveolar lavage fluid (BALF) and precision-cut lung slices (PCLS) were taken. An overview of the timeline of the experiment is given in Figure 1 and is further described below.

One week before expected parturition the sows were moved to farrowing pens, in which they were housed in crates. The expected parturition day was referred to as day 0 for all piglets, as sows were inseminated on the same day. Piglets from four sows were subjected from the first day of life onwards to conventional housing, while four other litters were exposed to enriched housing. Conventional pens were 5 m^2^ with partly slatted (50%) and party solid (50%) floor. In the conventional pens two chains were added. Enriched pens were 10 m^2^ with partly slatted (50%) and partly solid (50%) floor and a rooting area of 2.35 m^2^ in which straw, wood shavings (coarse and fine), two jute bags and branches of a broom were provided. To minimize differences in hygienic state between housing systems, straw and wood shavings were replenished daily (0.5 kg/day straw, 23 L/day of wood shavings), and on a weekly basis new branches of a wooden broom, new jute bags and 20 L of peat were added to the enriched pens. All pens were cleaned daily by removing faces and rinsing the slatted floors with water. All enrichment materials were sterilized by *γ*-irradiation. A heating lamp for the piglets was provided in each pen during the first week after birth. On day 3 of age, the piglets received an ear tag, they were treated with ironject® 20%+B12 (Dopharma, Raamsdonksveer, the Netherlands) and Baycox (Bayer, Animal Health), and tails were shortened according to standard procedures on the farm. The male piglets were not castrated.

All enriched and conventional pens had two drinking nozzles, one for the sow and one for the piglets. Sows were fed a standard commercial diet twice a day. The piglets received solid food *ad libitum*, starting at day 3. Lights were on between 7:00 am and 9:00 pm. Temperature was kept at 25°C during the first week after birth, and it was decreased by 1°C every week until it reached 22°C, the week before weaning.

From day 15 until weaning, the panels between two enriched pens with adjacent rooting areas were removed, allowing piglets from two different litters to mingle. Thus, the individual enriched pens of 10 m^2^ were temporarily transformed into pens of 20 m^2^ with a twice as large rooting area to enable early social interaction between enriched litters.

On day 28, the piglets were weaned and moved to weaner pens. Twenty-four piglets from conventional farrowing pens where placed in conventional pens (CCH), 24 piglets from conventional farrowing pens were placed in enriched pens (CEH), and 24 piglets from enriched farrowing pens were placed in enriched pens after weaning again (EEH). All piglets within the three housing treatments were equally divided over two pens, resulting in a total of six pens with 12 piglets each from four different litters. The piglets were equally mixed taking sex and body weight into account in order to obtain experimental subgroups with comparable composition. Temperature at weaning on day 28 was 28°C and was decreased by 2°C each week until it reached 22°C. Temperature was kept at 22°C from day 42 onwards. Body weights of all piglets was measured weekly from the day of parturition until the end of the experiment. On day 3 after birth an additional weighing moment took place.

On day 48, fourteen pigs (7 male/7 female) per treatment (*n* = 42 in total) were randomly selected to be used in the infection study, i.e., 10 pigs were selected for the dual infection studies and four pigs were used as controls and transferred to (4 km distance) the animal experiment facilities, suitable for infection studies with large animals in High Efficiency Particulate Air (HEPA) filtered animal rooms. Of each housing treatment (CCH, CEH, EEH) 10 pigs were placed in a pen where they kept the same housing enrichment, pen size, climate and access to feed and water. Additionally, of each housing treatment, four piglets were housed together in an semi-enriched pen which consisted of a weekly changed toy on a hanging chain and daily refreshed wood shavings (according to animal experiment regulations), as a control group. This control group was used to test if the co-infection caused the effect that was expected and not necessarily the housing effect.

On day 60 to 62, a total of eight piglet per housing treatment (four of each sex) were randomly selected from the remaining pigs on the farm and euthanized for the collection of BALF from the lungs and to sample lung tissue specimens for PCLS for *ex vivo* infection studies.

### 2.2 Infection procedure and inocula

To assess the effect of housing on disease susceptibility after infection, pigs were infected with PRRSV type 1 followed 8 days later by infection with *A. pleuropneumoniae* serotype 2. On day 60 (from here on referred to as infection day 0: ID0), CCH, CEH and EEH pigs (10 pigs per treatment) were inoculated intranasally with 2 mL inoculum containing 5.2 log_10_ 50% tissue culture infectious dose (TCID_50_) of PRRSV serotype 1 strain LV-Ter Huurne, deriving from the7th passage on porcine alveolar macrophages (PAMs) (28). Eight days after the viral infection (ID8), pigs were exposed to an aerosol infection with *A. pleuropneumoniae* serotype 2. The inoculum of *A. pleuropneumoniae* serotype 2 strain 17,415 (reference strain) was prepared as described earlier (29). In short, an aliquot of the reference strain, which was stored at −70°C, was thawed and the suspension was cultured overnight at 37°C under microaerophilic conditions on sheep blood agar plates (SB plates) with nicotinamide adenine dinucleotide (NAD). The next morning, colonies were transferred to fresh SB+NAD plates and cultured for 6 h. Thereafter colonies were rinsed from the plates and stored in phosphate buffer saline (PBS-13). The number of colony forming units (CFU) in the suspension was determined overnight and the inoculum diluted to achieve a concentration of 9 log_10_ CFU *A. pleuropneumoniae/*ml suspension. To expose pigs to an aerosol containing *A. pleuropneumoniae*, groups of four or five pigs were simultaneously kept for 30 min in a sealed aerosol chamber (110× 90 × 90 cm) with a controlled instream of air and controlled outstream of air through HEPA filters. The inoculum was administered via aerosol using the aerosol nebulizer Aeroneb Pro (EMKA Technologies, Paris, France). An amount of 5 mL of the inoculum suspension was administered during a period of 15 to 18 min and the amount of viable bacteria was measured by sampling aliquots of air in a six-stage Anderson stage sampler at 8 and 25 min after start of aerosol exposure. The procedures have previously been described (21, 30, 31).

The 12 control pigs underwent the same procedures as the infected animals at ID0 and ID8, however instead of PRRSV inoculum, 1.5 mL RPMI medium was used and instead of the *A. pleuropneumoniae* inoculum, 5 mL of PBS was used.

On day 71 (ID11, i.e., 11 days after PRRSV infection and 3 days after *A. pleuropneumoniae* infection), all 30 infected pigs and 12 control pigs were euthanized by injecting pentobarbital (Euthasol 40%, AST Farma) in the auricular vein, while they were restrained and thereafter exsanguinated.

### 2.3 Clinical examination

Rectal temperature (by Microlife® digital thermometers) was assessed once a day from 11 days before infection (ID-11, day 49) and twice daily from ID0 until ID11. At the days of PRRSV and *A. pleuropneumoniae* infection, temperature was measured prior to the infection procedure and 4 h after *A. pleuropneumoniae* infection procedure took place. All piglets were observed and inspected for respiratory symptoms (coughing, breathing problems, sneezing) twice per day (at 9:00 am and 4:00 pm) from ID0 until the end of the experiment (ID11).

### 2.4 Blood collection and analysis

From all 42 piglets (infected + control) blood samples were collected via jugular vein puncture on days 26 (2 days before weaning), 34 (6 days after weaning), 48 (just before transport), 56 (4 days before PRRSV infection), 63 (ID3), 65 (ID5), 67 (ID7), 69 (ID9) and 71 (ID11) (Figure 1).

#### 2.4.1 WBC count and differentiation

The total count of white blood cells (WBC) was determined in EDTA stabilized blood samples for all blood sampling days with an automated, impedance technology based hematology analyser (blood cell counter Sysmex pocH-100 iV diff, Kobe, Japan). WBC were differentiated into neutrophil granulocytes and monocytic blood cells, i.e., lymphocytes and monocytes.

#### 2.4.2 Phenotyping of blood cells

Phenotyping of WBCs was further performed in full blood samples by flow cytometry as previously described (21). The blood cells were incubated with a primary antibody-mixture of monoclonal antibodies (mAb) against CD3 (clone PPT3, IgG1), CD4 (Clone 74–12-4, IgG2b), and CD8α (clone 76–2-11, IgG2a) (all mAb’s were supplied by Southern Biotech) for triple labeling or for single labelling with mAb against CD172a (clone 74–22-15; IgG2b) (supplied by VMRD) or CD21 (clone B6-11C9) (Southern Biotech). Non-related isotype mAb’s (Southern Biotech) were used as controls. After labelling with the primary mAb’s, cell suspensions were incubated with PBS supplemented with 1% (v/v) pig serum and 0.1% (w/v) sodium azide, containing isotype specific secondary antibodies (goat anti–mouse IgG1-APC, IgG2b-FITC and IgG2a-PE; Southern Biotech). Blood were analyzed on a FACSVERSE™ (BD Biosciences) using the BD FACSsuite™ software. The flow cytometry data were analyzed with Flowjo™ software version 10.0.

Blood cells were first gated on the basis of forward-scatter (FCS) versus sideward scatter (SSC) diagram as described by Nielsen et al. (32) and then analyzed according to their antigen marker profile. Based on the WBC counts in blood by the automated blood cell counter the absolute numbers of the different phenotypes were calculated. In blood neutrophils were identified in blood by an SSC^high^ CD172a^+^ profile. T-cells were distinguished in the lymphocyte gate by the following marker combinations: naïve/non-activated T-helper cells: CD3^+^CD4^+^CD8-, cytotoxic T-cells: CD3^+^CD4^−^CD8^+^, memory/activated T-helper cells: CD3+ CD4+CD8+ and natural killer (NK) cells: CD3-CD4-CD8+ [48, 49]. Monocytes were identified in blood by low granularity, i.e., SSC^low^CD172a^+^.

#### 2.4.3 Whole blood stimulation assay

To investigate immune responsiveness of white blood cells after different housing treatments Heparin-treated whole blood samples were obtained on day 26, 34 and 48. The whole blood was stimulated with lipopolysaccharide (LPS), and levels of cytokine production (IL1ß, IL-6, TNF-*α*) were quantified by ELISA. The results of the WBC counting were used to dilute the blood with RPMI 1640 medium Glutamax with 5% foetal calf serum and 1% penicillin/streptomycin to obtain a WBC concentration of 5 × 10^6^ cells/ml. Then 0.5 mL of such diluted blood was transferred to 48-well plates, and cells were either stimulated by adding 50 μL LPS/PBS solution (*Escherichia coli*, Sigma-Aldrich Chemie N.V., NL), i.e., 1 μg LPS/ml, or 50 μL PBS only. Cells were incubated for 20 h before culture medium was harvested and stored at −80°C until performing cytokine analysis. The cytokines were determined by using the commercially available ELISA kits Duoset™ (R&D Systems) for IL1ß and IL-6, and ELISA kit Quantikine™ (R&D Systems) for the analysis of TNF-*α* according to the manufacturer’s instructions. Cytokine concentration in incubations with only PBS (controls) were subtracted from corresponding concentrations in incubations with LPS to determine cytokine response.

#### 2.4.4 Detection of viral RNA in serum

A quantitative reverse transcription polymerase chain reaction (qRT-PCR) was performed on RNA isolated from blood serum, sampled at ID-4, ID0, ID3, ID5, ID7, ID9 and ID11. A one-tube qRT-PCR was performed with the Applied Biosystem 7,500 Fast System instrument using the Quantitect Probe RT-PCR kit from Qiagen. The reaction mixture (25 μL) contained 0.25 μL of kit-supplied enzyme, 12.5 μL of Quantitect Mix, 15 μM of each primer (Fw: 5′- GAT GAC RTC CGG CAY C -3′, Rev.: 5′- CAG TTC CTG CGC CTT GAT −3′) and 10 μM of probe (5′- Fam-TGC AAT CGA TCC AGA CGG CTT-Tamra- 3′). RT-PCR was performed at 30 min at 50°C and 15 min at 95°C followed by a two-step cycling protocol: 94°C for 20s, and 55°C for 45 s for 40 cycles. Analysis was performed with 7,500 Software v2.0.6 (Applied Biosystems). Viral RNA concentration (expressed as TCID50 equivalents per g) of each serum sample was calculated using a standard curve, constructed by extracting RNA from five decimal dilutions of medium spiked with known concentrations of infectious virus (33).

### 2.5 Detection of typical *Actinobacillus pleuropneumoniae* lesions and histological assessment of the lungs

After the pigs had been euthanized, special attention was paid during necropsy to patho-morphological changes in the respiratory tract. The type and size of lung lesions were recorded in a lung drawing and the proportions of the affected lung surface was calculated. Necro-haemorrhagic lesions, typical for *A. pleuropneumoniae*, were macroscopically detected and histologically confirmed. Total number of pigs with these lesions, were counted and presented as total number and percentage of pigs with typical *A. pleuropneumoniae* lesions.

For histological assessment of the lungs, 6 tissue samples per pig from predefined locations in the lungs (cranial, cardial and caudal lobe of the left and right lobe) were formalin-fixed, processed and embedded in paraffin. Tissue sections were stained with Haematoxylin-Eosin (HE) and a semi-quantitative, patho-histological assessment of HE stained slides encompassed the extent of pneumonia throughout the predefined locations in the lungs. Patho-histological assessment included 4 features: (1) the presence of focal or diffuse alterations with interstitial or catarrhal pneumonia or atelectasis, (2) the extent of infiltration of alveolar septae with mononuclear cells (3) the extent of infiltration of mononuclear cells in the perivascular/peribronchiolar area and (4) pleuritis. A histological score of 0 to 3 was used to describe the severity of changes per feature (i.e., 0 = no findings, 1 = mild focal manifestation, 2 = moderate, multifocal manifestation or diffuse manifestation, 3 = severe diffuse manifestation). During histological examination, the pathologist was blinded with regard to treatment (Housing and Infection). The scores from all 6 slides per lung were added to obtain an overall histology score, which could add up to a maximum of 72 points per pig (6 slides per pig, 4 histological features, and maximum score of 3 per feature). The total exudative and interstitial components score per pig was also calculated and presented separately as these changes in the lungs are considered to be related to PRRSV infection (34–36). This score could reach a maximum of 18 (6 slides per lung, 1 histological feature, and maximum score of 3).

#### 2.5.1 Re-isolation of *Actinobacillus pleuropneumoniae*

For re-isolation of *A. pleuropneumoniae*, two lung tissue samples of predefined lungs locations (cranial lobe, caudal dorsal lobe) and samples of bronchial lymph nodes were taken. When a macroscopic visible lung lesion was present at another location than the previous described locations, a third sample was taken. The collected lung and bronchial lymph node tissues were prepared, grinded and plated on SB+NAD plates as described by Jirawattanapong et al. (37) and Kamp et al. (38). To confirm that colonies were *A. pleuropneumoniae* serotype 2, dependency for NAD was tested by comparing growth, on SB and SB+NAD plates followed by agglutination with serotype 2-specific hyper-immune rabbit antiserum (37).

### 2.6 Broncho-alveolar lavage fluid

Eight pigs per treatment (total *n* = 24) were euthanized (as described above) on either day 60, 61 or 62 (eight per day). From these animals BALF and PCLS were taken (see below). BALF was obtained from the right cranial lung lobe during necropsy as previously described (21), followed by isolation and phenotypic characterization of broncho-alveolar neutrophil granulocytes and monocyte/macrophages as described before (21). In summary, the following stainings were performed: CD172, CD163, CD14 (clone MIL2, isotype IgG2b, BioScource) and TLR4 (Isotype IgM, generous gift by J. Dominguez). The following combinations of secondary antibodies (SoutherBioTech, US) and fluorochromes were used: IgG1-APC, IgG2b-FITC and IgG2a-PE. The following possible populations of macrophages in BALF were calculated separately: all macrophages with expression of CD172+ or TLR4+ or CD14+, and with the following co-expressions: CD14+TLR4+, CD14+TLR4−, CD14−TLR4+, CD172+TLR4+, CD172+TLR4−, CD172−TLR4+, CD172+CD14+; the proportion of neutrophil granulocytes was calculated by the expression of CD172a^+^CD163^−^ staining.

### 2.7 *Ex vivo* studies with precision-cut lung slices

To investigate the impact of enrichment on the local response in lungs to infection *ex vivo* studies were performed on PCLS of lung tissues of the uninfected pigs that were euthanized at 60–62 days of age. PCLS were produced from lungs of 6 animals per treatment condition (total *n* = 18). PCLS were prepared from agarose filled right cranial lung lobes (39). The embedded lung tissue was subsequently cut into slices of 350 μm and incubated for 24 h with swine influenza A, *A. pleuropneumoniae* or a combination of both pathogens. The PCLS were incubated first 2 h in 24-well plates with one mL of medium [RPMI 1640 medium (Life Technologies)] with 5% AB11 (1.0 g/L streptomycin; 0.02 g/L amphotericinum B; 0.5 g/L polymyxcin B; 2.4 g/L kanamycin sulfate in Hanks BSS (Wageningen Bioveterinary Research, Lelystad, the Netherlands), 100 U/mL penicillin (Gibco) and 100 μg/mL streptomycin (Gibco) at 37°C and 5% CO2. After 2 h the PCLS culture medium was replaced by medium without antibiotics and infected either with swine influenza strain A/Sw/Oedenrode/96 (H3N2) (4*10^5^ PFU/mL), or *A. pleuropneumoniae* serotype 2 (2.5*10^6^ CFU/mL) only or in case of a combined infection starting with an infection with swine influenza followed after 3 h by washing and replacing medium and adding *A. pleuropneumoniae*. Thereafter plates were cultured for 24 h at 37°C with 5% CO_2_. Then, the remaining RPMI medium was removed, the slides were washed one time with RPMI medium and the 24-well plates were frozen at −80°C. In a following step RNA was extracted as described (40), subjected to DNAase treatment using a Direct-zol™ RNA MiniPrep (BaseClear Lab Products, Leiden, the Netherlands) according to the manufacturer’s instructions and cDNA was made using random hexamer primers and reverse transcriptase. PCR assays were conducted with online detection using the Syber Green PCR Master Mix (Applied Biosystems) in an ABI 7500 Real-Time PCR system (PE Applied Biosystems, Foster City, CA, USA). PCR assays were performed to quantify expression of the following genes: endogenous gene GAPDH (GAPDH Fw: TGCCAACGTGTCGGTTGT; Rv: TGTCATCATATTTGGCAGGTTTCT), target genes: interferon beta (IFN-*β* Fw: AGTGCATCCTCCAAATCGCT; Rv: TCTCCTCAGGGACCTCAAAGTTC), interleukin 8 (IL-8 Fw: TTCGATGCCAGTGCATAAATA; Rv: CTGTACAACCTTCTGCACCCA) and Toll-like receptor 4 (TLR4 Fw: GCCATCGCTGCTAACATCATC; Rv: CTCATACTCAAAGATACACCATCGG). The relative gene expression of target genes to endogenous gene was performed using the non-infected controls of treatment groups as reference for expression fold change and analysis was performed by the 2^-ddCt^ method according to (41).

### 2.8 Statistical analysis

Statistical analyses were performed with SAS (SAS 9.3, SAS Institute Inc.). For all data, except the occurrence of lung lesions (see below), mixed linear models were used. Pairwise comparisons (>3 groups of means) were adjusted by Tukey corrections. Results are presented as means ± standard deviation. If needed, variables were square root or log transformed to obtain normally distributed residuals.

#### 2.8.1 Before infection

WBC counts and growth before infection were analyzed with day as repeated effect using the repeated statement of SAS with the autoregressive covariance structure. Housing (CCH, EEH or CEH), day (26, 34, 48, 56 in case of WBC; 3, 6, 13, 20, 27, 34, 41, 55 in case of body weight) and their interaction were used as fixed effects and sow as random effect. Cytokine profiles in the WBSA after LPS stimulation analyzed at three specific timepoints (day 26, 34, and 48). Housing (CCH, CEH, EEH) was included as fixed effect and sow as random effect.

#### 2.8.2 After infection

To check whether the infection was successful, growth and average rectal temperature were compared between infected pigs and controls using infection (yes vs. no) as fixed and sow as random effect.

Since infection was successful (see results), viral RNA levels, WBC counts, growth and rectal temperature were analyzed for infected animals only. These time dependent variables were analyzed with day (or timepoint in case of rectal temperature) as repeated effect at the pig level using the repeated statement of SAS with the autoregressive covariance structure. Housing (CCH, CEH, EEH), day (or timepoint in case of rectal temperature) and their interaction were used as fixed effects and sow as random effect. To investigate viral RNA in more detail, additional mixed models were run per separate treatment, with day included as fixed effect (ID0 excluded). For rectal temperature, separate analyses were done for the period from 2 days before PRRSV infection (ID-2) until the time point just before *A. pleuropneumoniae* infection [ID8(1)], and the period from *A. pleuropneumoniae* infection [ID8(1)] until the end of the experiment (ID11) to evaluate the effects of PRRSV and *A. pleuropneumoniae* separately. Analysis revealed that body weight did not differ between infected pigs in different housing groups both before and after infection (data not shown).

Variables measured at a single specific time point (i.e., histology scores, BALF cells and PCLS) were analyzed with housing (CCH, CEH, EEH) as fixed effect and sow as random effect. Cytokine productions in the WBSA were measured at three different days, and all three measurement days were analyzed separately. Effects of housing treatment on the occurrence of typical *A. pleuropneumoniae* lung lesions (i.e., lung lesions present or absent) and on the occurrence of re-isolated *A. pleuropneumoniae* were analyzed with a generalized mixed linear model (GLIMMIX procedure in SAS), with a logit link and binary distribution with housing as fixed effect and sow as random effect. Here, odds ratios (OR) are presented to indicate the effect size.

## 3 Results

### 3.1 Effects of enrichment on pig health around weaning

No clinical signs of disease were observed in any of the pigs during suckling and nursery phase.

#### 3.1.1 Whole blood cell counts and cell types

**Figure 2:**
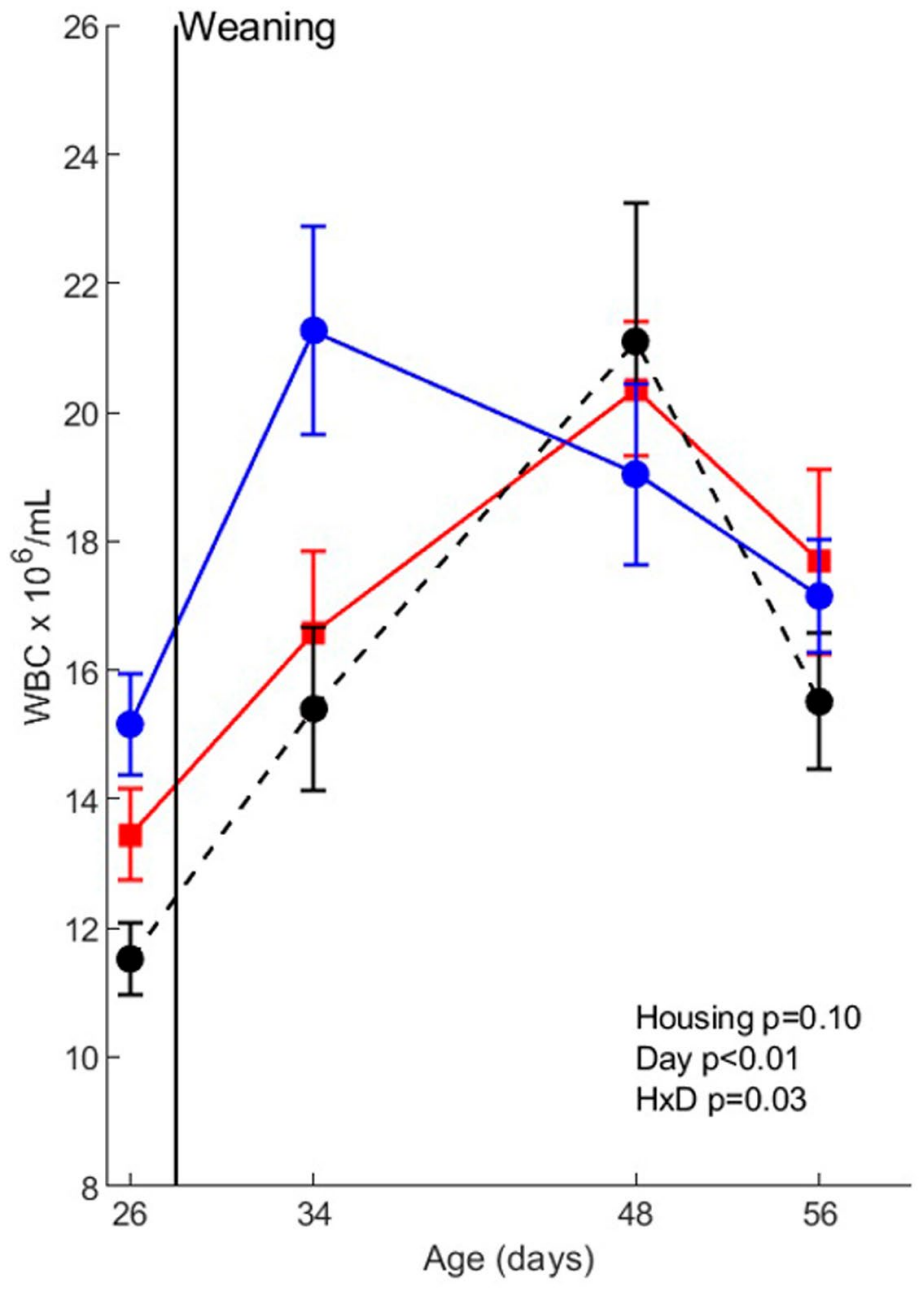
Whole blood cell (WBC) counts before co-infection in pigs (*n* = 30) with different housing treatments (red = CCH (conventional-conventional housing); black = CEH (conventional-enriched housing); blue = EEH (enriched-enriched housing)). HxD: housing-day interaction effect.

The effects of housing treatments (without infection) on white blood cell numbers and white blood cell composition are shown in Figure 2. WBC counts were affected by day (age) (*p* < 0.01) and housing x day (*p* = 0.03), indicating a different time course of the WBC counts around weaning. Post-hoc analysis revealed that in EEH pigs WBC counts increased from day 26 (before weaning) to day 34 (after weaning), with levels on day 48 and 56 in between. In CCH pigs, WBC counts were higher on day 48 than on day 26, whereas in CEH pigs WBC counts were higher on day 48 than on all other days.

**Figure 3:**
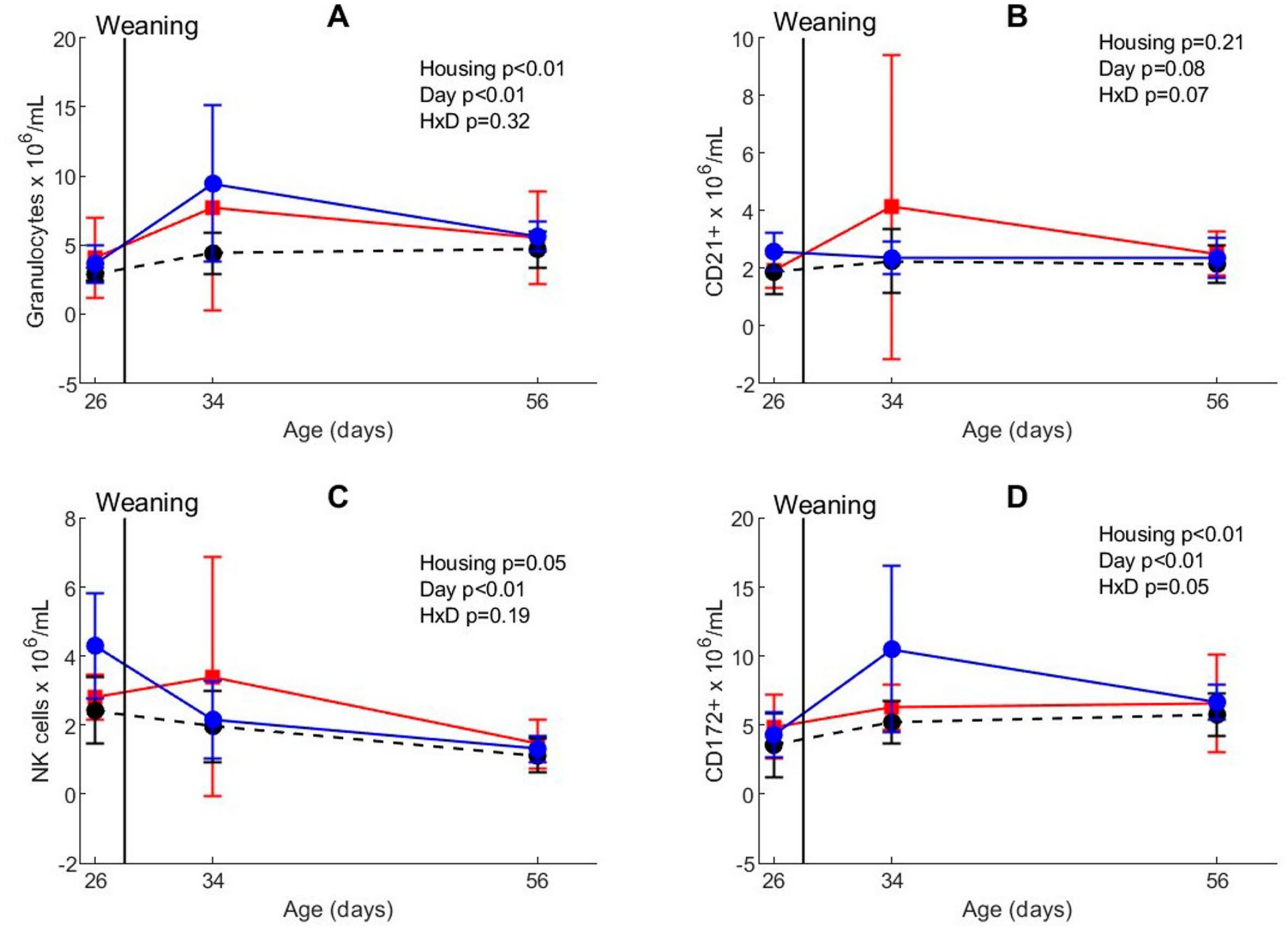
Blood phenotyping in absolute numbers for **(A)** neutrophilic granulocytes, **(B)** CD21+ B, **(C)** NK, and **(D)** CD172+ cells in pigs (*n* = 30) with different housing treatments [red = CCH (conventional-conventional housing); black = CEH (conventional-enriched housing); blue = EEH (enriched-enriched housing)] before co-infection. HxD: housing-day interaction effect.

Whole blood analyses revealed that the mobilization of white blood cells was affected by weaning and different housing treatments (Figure 3; Supplementary Table S1). The absolute number of neutrophil granulocytes was higher after weaning than before weaning (day effect, *p* < 0.01). Housing treatment also affected granulocyte numbers (*p* < 0.01), with less granulocytes in CEH than in CCH and EEH. The number of neutrophils and monocytes (CD172+ cells) increased directly after weaning (day effect, *p* < 0.01), but the response depended on housing (housing x day interaction *p* = 0.048), with higher numbers on day 34 in EEH than CCH and CEH pigs. The number of NK cells gradually decreased between all timepoints (day effect, *p* < 0.01), and CEH pigs tended to have less NK cells than CCH and EEH pigs (housing effect, *p* = 0.05). In CCH pigs the numbers of CD21+ B-cells after weaning tended to be higher on day 34 compared to day 26, whereas in EEH and CCH pigs the numbers of CD21+ B-cells tended to stay on a same level (housing x day interaction *p* = 0.07).

#### 3.1.2 Whole blood stimulation assay by LPS stimulation

**Figure 4:**
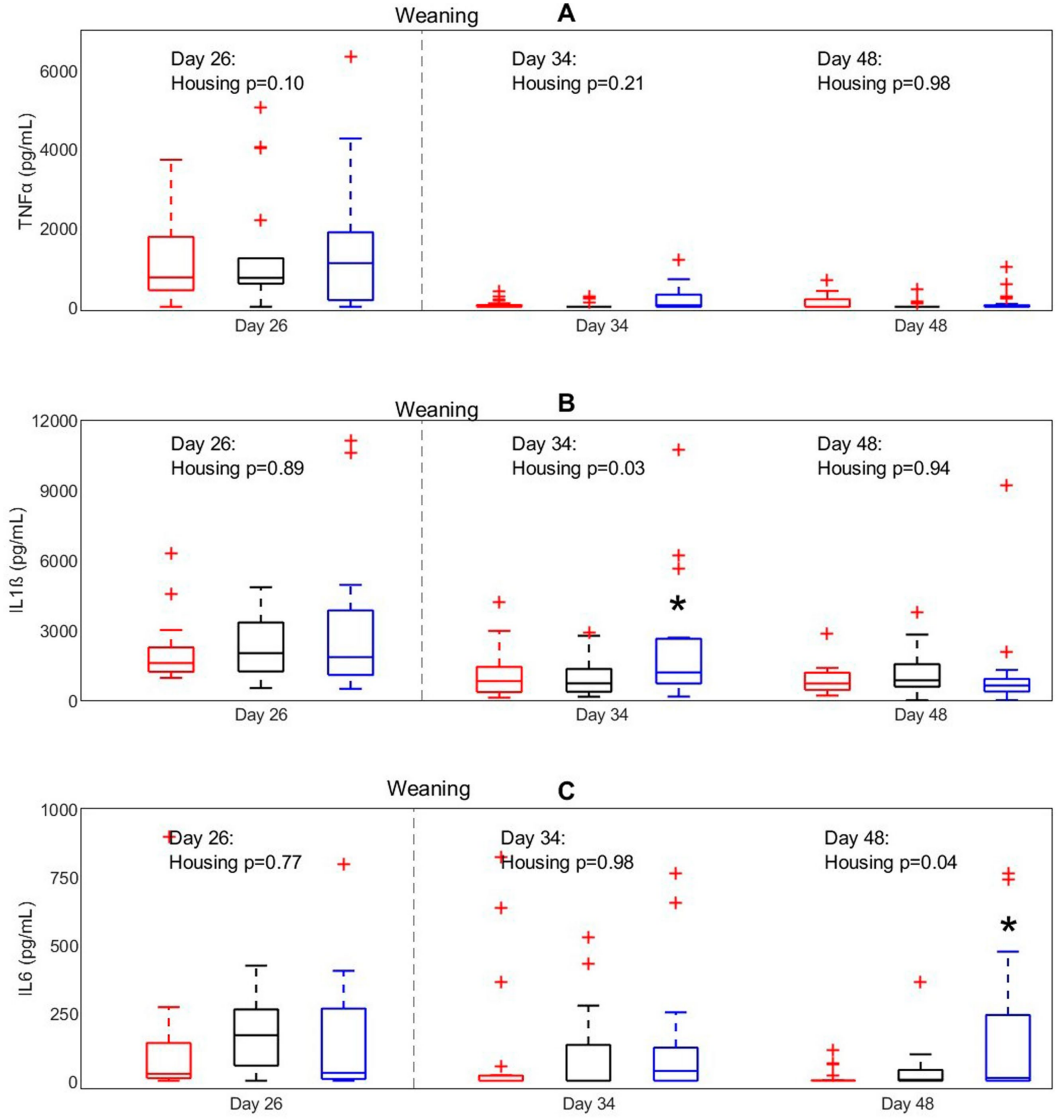
Concentrations of **(A)** TNFα, **(B)** IL-1ß, and **(C)** IL-6 after LPS stimulation in pigs (*n* = 30) with different housing treatments [red = CCH (conventional-conventional housing); black = CEH (conventional-enriched housing); blue = EEH (enriched-enriched housing)]. ^*^ Housing treatment significantly differs from the other housing treatments on the same day (*p* < 0.05).

To investigate the effect of housing on inflammatory responsiveness of white blood cells before and after weaning an *in-vitro* WBSA with a LPS challenge was performed (Figure 4). Housing treatment affected IL1ß concentrations after *in vitro* LPS stimulation on day 34 (*p* = 0.03), with higher concentrations for EEH pigs than for CCH and CEH pigs on this day. IL6 concentrations on day 48 were affected by housing (*p* = 0.04), with also higher concentrations for EEH pigs compared with CCH and CEH pigs.

#### 3.1.3 BALF immune cell phenotyping

**Figure 5:**
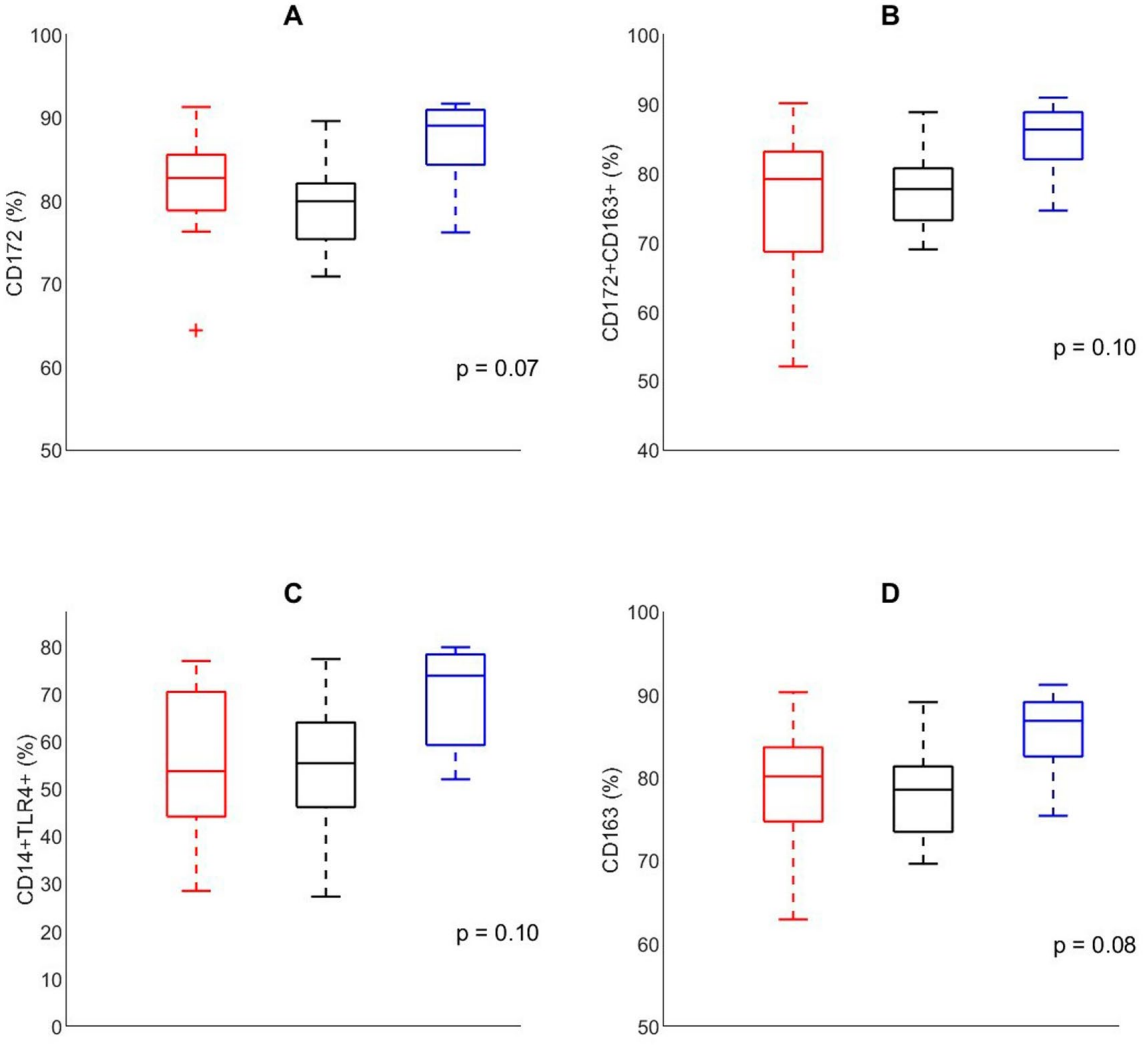
Percentage of cells expressing **(A)** CD172, **(B)** CD172^+^CD163^+^, **(C)** CD14^−^TLR4^+^, **(D)** CD163, in broncho-alveolar lavage fluid in control pigs (*n* = 24) on day 60 with different housing treatments [red = CCH (conventional-conventional housing); black = CEH (conventional-enriched housing); blue = EEH (enriched-enriched housing)].

To evaluate the effects of housing on lung immune function prior to infection, cellular composition of the BALF was analyzed in pigs that were not involved in the infection trial at D60 (Figure 5). The composition of BALF tended to differ between different housing groups. The relative number of CD 172+ macrophages tended to be affected by housing (*p* = 0.08), being higher in EEH pigs compared with CCH and CEH pigs. Similar effects of housing treatment were found for the relative number of CD163 macrophages (*p* = 0.08) and CD172+CD163+ macrophages (*p* = 0.10), where EEH pigs tended to have a higher proportion of CD163 and CD172+163+ macrophages compared with CCH and CEH pigs. Also macrophages expressing CD14+/TLR4+ tended to be most numerous in the EEH pig group (housing effect, *p* = 0.10).

### 3.2 *Ex vivo* assessment of enrichments effects on immune response in lung tissue

**Figure 6:**
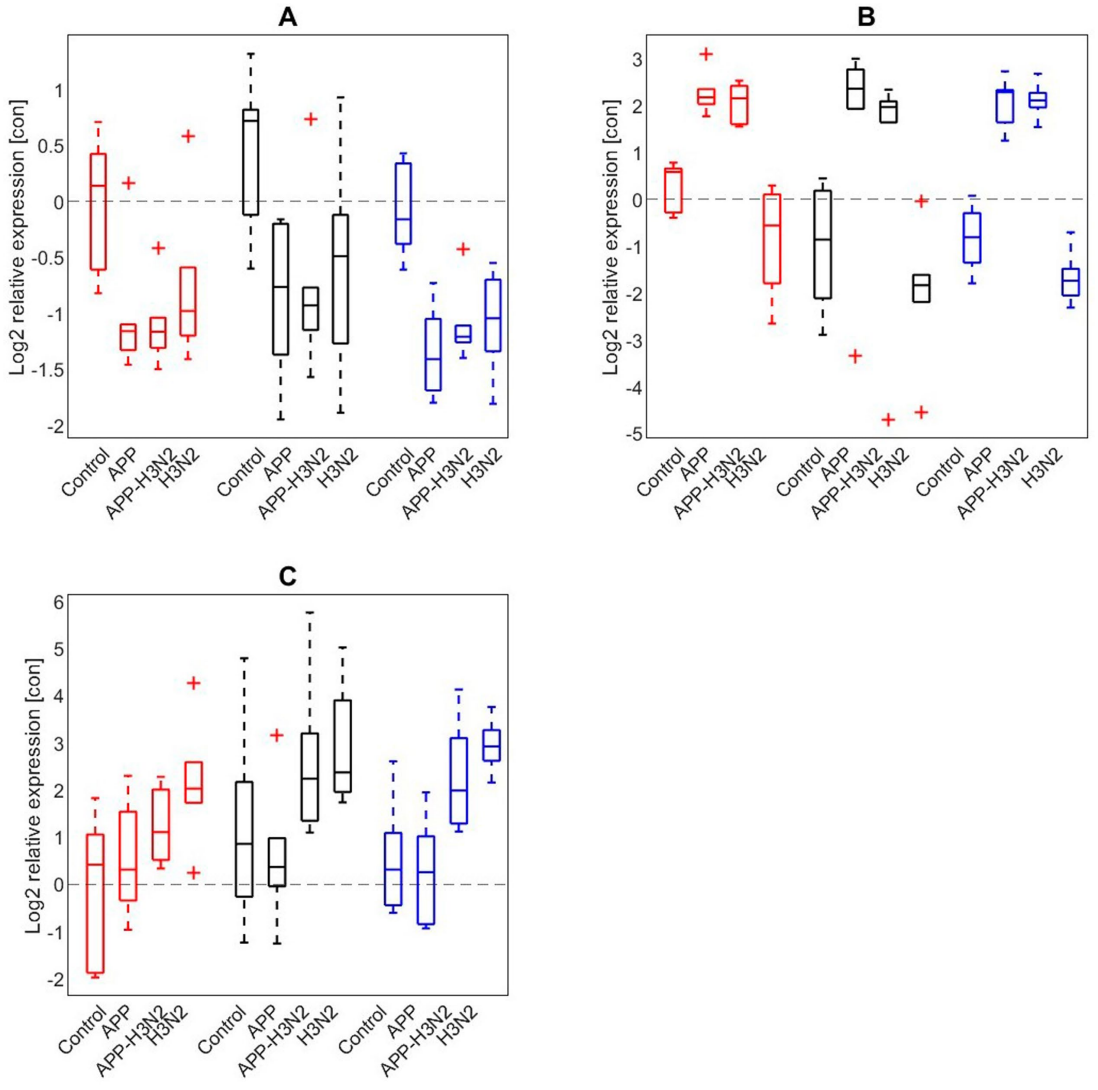
Log2 relative expression of **(A)** TLR4, **(B)** IL-8, **(C)** IFN-*β* after infection of precision cut lung slices without infection (control), or infected with *Actinobacillus pleuropneumoniae* (APP), APP and swine influenza (H3N2), or H3N2 alone in CCH (conventional-conventional housing; red), CEH (conventional-enriched housing; black) and EEH (enriched-enriched housing; blue) pigs (*n* = 18).

*Ex vivo* PCLS experiments were performed to study the effects of enrichment treatment on mRNA responses of IL-8, IFN-*β* and TLR-4 in lung tissues to infections with *A. pleuropneumoniae*, swine influenza (SwIAV H3N2) or a combination of these (Figure 6). The relative expressions of IL-8, IFN- β and TLR-4 did not significantly differ between treatment groups (lung tissue was derived from either the CCH, CEH or EEH group). Investigating the relative expressions of IL-8, IFN-*β* and TLR-4 across *ex vivo* treatment groups in more detail showed that the relative gene expression of TLR-4 in the lung slices was lowered in all *ex vivo* treatment groups after single and combined infection compared to the non-infected control slices, but with no differences between treatment groups. The relative expression of IL-8 in lung slices increased after *A. pleuropneumoniae* stimulation with or without SwIAV H3N2 over all three *ex vivo* treatment groups when compared with the non-infected control slices. The relative expression of IFN-*β* was highest after SwIAV H3N2 single infection compared with the non-infected control slices in all *ex vivo* treatment groups. Combined infections with SwIAV H3N2 and *A. pleuropneumoniae* led to higher relative IFN-β gene expression than the non-infected control slices.

### 3.3 Disease outcome after co-infection with PRRSV and *Actinobacillus pleuropneumoniae*

After the PRRSV-infection, no clinical signs of disease were observed in the CCH, CEH and EEH groups [with the exception of coughing in one pig of the EEH group once at ID4(2)]. On the day of *A. pleuropneumoniae* infection at ID8(2) pigs in the EEH and CEH groups showed reduced appetite, activity and respiratory distress. From the day after infection with *A. pleuropneumoniae* (ID9) mild clinical signs of disease were observed at one or two of five observation moments consisting of reduced appetite (1/10 CCH group, 1/10 EEH group), a mildly reduced activity (3/10 CCH, group, 2/10 CEH group, 4/10 EEH group) and/or a moderate respiratory distress (2/10 CCH group, 1/10 EEH group).

#### 3.3.1 Rectal temperature

**Figure 7:**
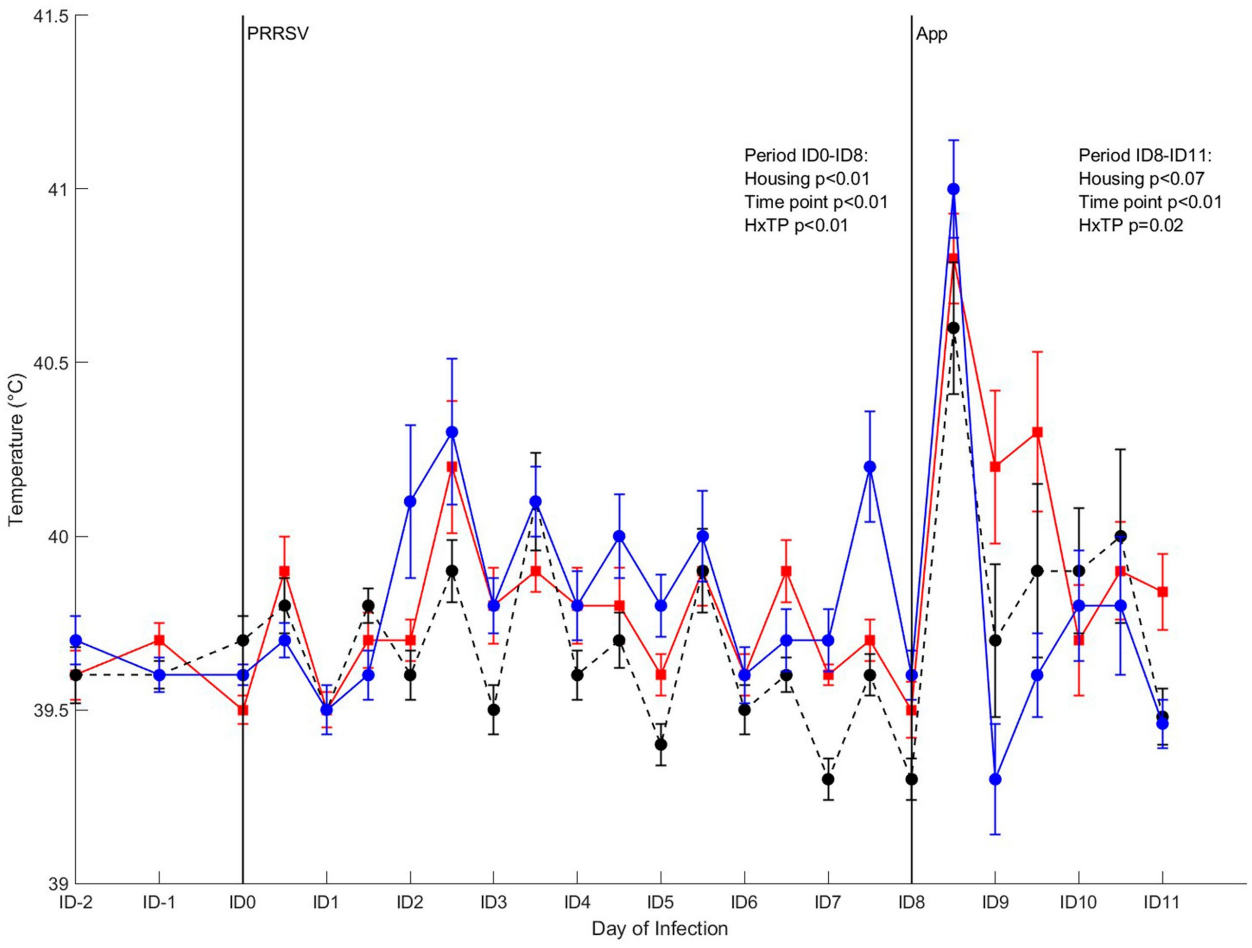
Rectal temperature in pigs (*n* = 30) with different housing treatments [red = CCH (conventional-conventional housing); black = CEH (conventional-enriched housing); blue = EEH (enriched-enriched housing)] after co-infection with Porcine Reproductive and Respiratory virus (PRRSV) and *Actinobacillus pleuropneumoniae* (APP). Infections at infection day (ID)0 and ID8 with PRRSV and APP are indicated as black vertical lines. HxTP: housing-timepoint effect.

Rectal temperature of the infected pigs from 2 days before infection with PRRSV to 8 days after infection (ID8) was affected by housing (*p* < 0.01), timepoint (*p* < 0.01) and their interaction (*p* < 0.01; Figure 7). Post-hoc analysis revealed that the time course of the rectal temperature varied over time between treatments, and on some timepoints significant differences between treatments were present. Mean rectal temperature of CCH and EEH pigs showed a fever peak at the second measurement of the second day after infection [ID2(2)], whereas for CEH this peak was seen on ID3(2). As a result, CEH pigs had a lower rectal temperature on ID2(2) than CCH and EEH pigs. In EEH pigs fever occurred on ID2(1) and stayed until ID3(2), followed by subfebrile body temperatures for three additional days. In the CCH and CEH groups rectal temperatures stayed in normal ranges. The infection with *A. pleuropneumoniae* induced increased rectal temperatures at 4 h after infection in all three groups. In CEH and EEH pigs rectal temperature dropped on ID9(1), whereas in CCH pigs this happened on ID10(1). As a result, at ID9(1), rectal temperatures of CCH pigs were higher than those of EEH pigs, with levels of CEH pigs in between.

#### 3.3.2 WBC counts and differentiations

**Figure 8:**
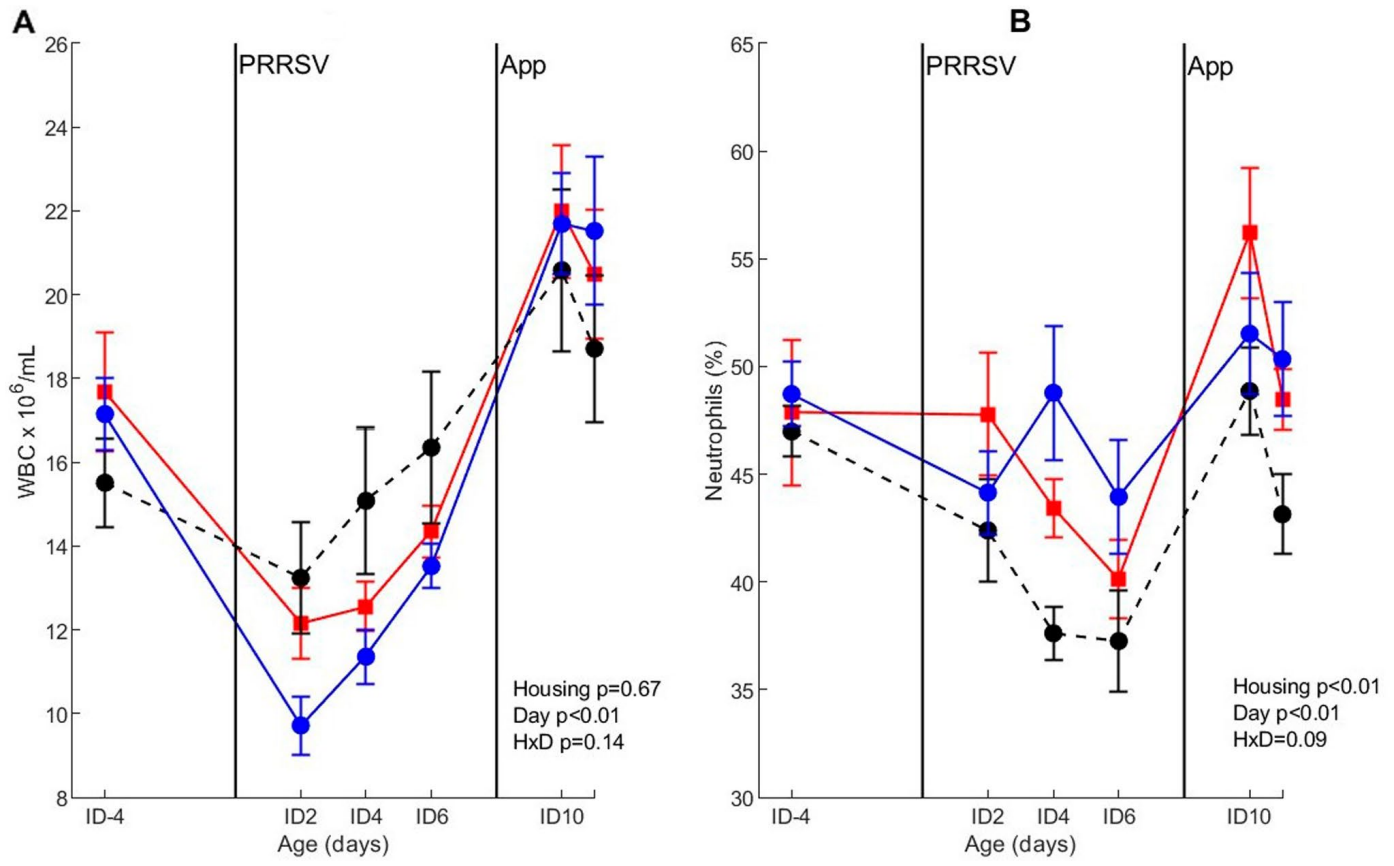
Whole blood cell count (WBC) **(A)** and percentage of neutrophils **(B)** after co-infection with PRRSV and *Actinobacillus pleuropneumoniae* (APP) in pigs (*n* = 30) with different housing treatments [red = CCH (Conventional-conventional housing); black = CEH (conventional-enriched housing); blue = EEH (enriched-enriched housing)]. Infections at infection day (ID) 0 and ID8 with Porcine Reproductive and Respiratory virus (PRRSV) and APP are indicated as black vertical lines. HxD: housing-day interaction effect.

For WBC counts after co-infection, only a day-effect (*p* < 0.01) was found, where WBC counts dropped on ID2 and ID4, and increased to near pre-infection levels on ID6 (irrespective of housing treatment). After *A. pleuropneumoniae* infection on ID8, WBC counts increased at 2 days after infection in all treatment groups. The percentage of neutrophils after co-infection was affected by housing (*p* < 0.01) and day (*p* < 0.01), where neutrophil percentage on ID-4 was higher than on day ID6. CEH pigs had a lower percentage of neutrophils than CCH and EEH pigs (Figure 8). For all housing treatments, percentage of neutrophils increased after *A. pleuropneumoniae* infection, and was highest on ID10 after which it declined again on day ID11.

#### 3.3.3 Viraemia

**Figure 9:**
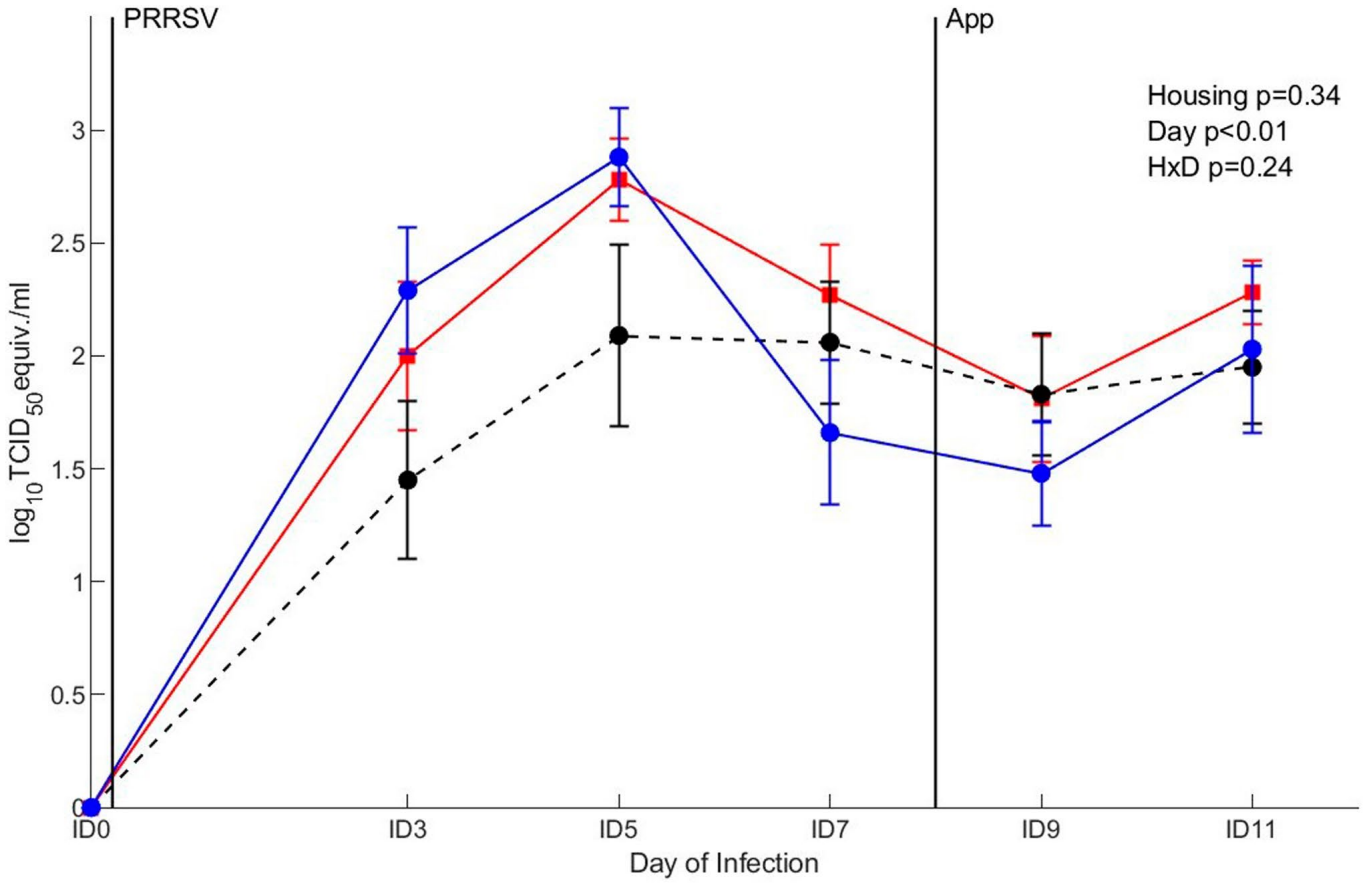
qRT-PCR in serum after co-infection with Porcine Reproductive and Respiratory virus (PRRSV) and *Actinobacillus pleuropneumoniae* (APP) in pigs (*n* = 30) with different housing treatments [red = CCH (conventional-conventional housing); black = CEH (conventional-enriched housing); blue = EEH (enriched-enriched housing)]. Infections at infection day (ID) 0 and ID8 with PRRSV and APP are indicated as black vertical lines. HxD: housing-day interaction effect.

The clearance of viral RNA was assessed after co-infection with PRRSV and *A. pleuropneumoniae.* The viral RNA expressed as log10 TCID_50_ eq/mL was affected by day (*p* < 0.01), where the presence of virus in serum gradually increased between ID0 and ID5, after which it declined between ID5 and ID7 and remained on a stable level (Figure 9). Overall, viral load was not affected by housing or the interaction between housing and day. To investigate the viral RNA over the course of infection in more detail, virus RNA was analyzed per separate treatment. A significant mean virus reduction of 1.2 log_10_ TCID_50_ eq/mL log_10_ was measured in pigs of the EEH group from ID5 to ID7 (*p* < 0.01), whereas virus reduction in the other groups was not significant between these timepoints. No differences in viral RNA between other time points were found for all treatments.

#### 3.3.4 Effect of *Actinobacillus pleuropneumoniae* infection on lungs

**Figure 10:**
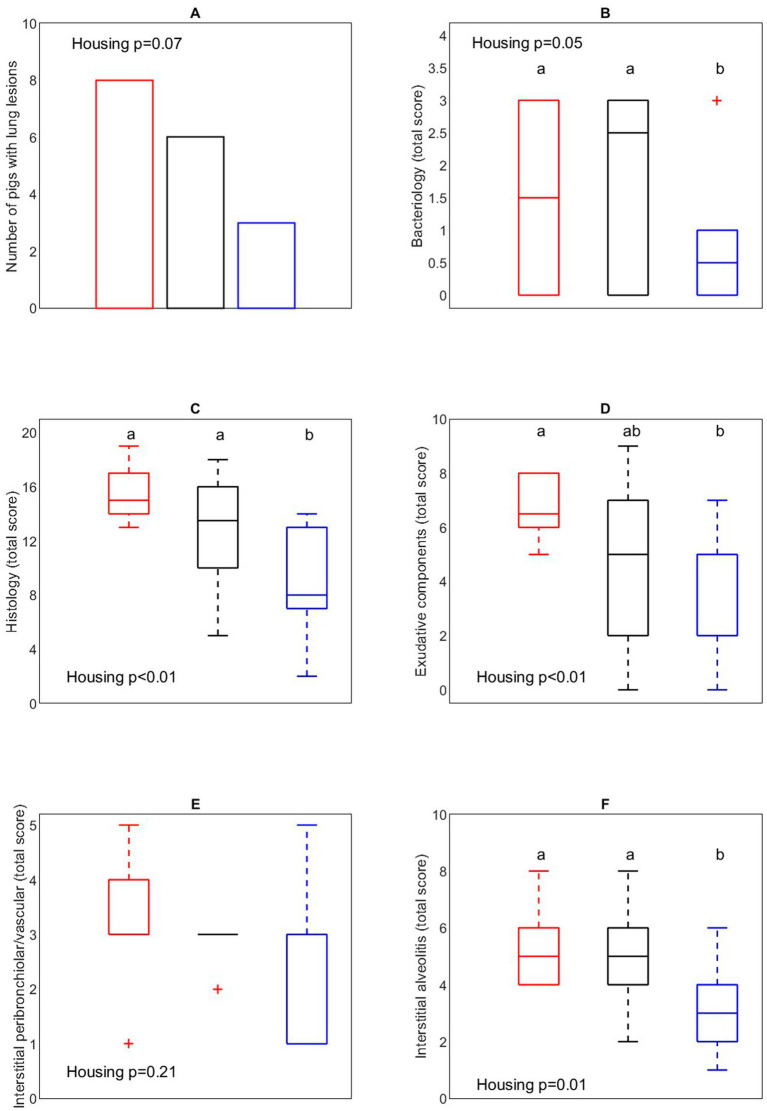
Number of pigs with lung lesions **(A)**, bacteriological **(B)**, overall histology **(C)**, exudative component **(D)**, interstitial peribronchiolar/vascular component **(E)**, interstitial alveolitis component **(F)** score of lung tissue in pigs (*n* = 30) with different housing treatments [red = CCH (conventional-conventional housing); black = CEH (conventional-enriched housing); blue = EEH (enriched-enriched housing)] after co-infection with Porcine Reproductive and Respiratory virus (PRRSV) and *Actinobacillus pleuropneumoniae* (APP). ^a,b^Treatments lacking a common letter significantly differ (*p* < 0.05).

To address the impact of housing on the outcome of the *A. pleuropneumoniae* co-infection, occurrence of typical (multi)focal necrohaemorrhagic pleuro-pneumonia lesions were examined (Figure 10A). An overall tendency (*p* = 0.07) for differences in proportion of pigs with lung lesions between housing treatments was found, where *post hoc* comparison showed that more CCH pigs (8 out of 10) developed lung lesions after PRRSV and *A. pleuropneumoniae* infection compared with EEH pigs (3 out of 10) (OR = 9.33, *p* = 0.03). From the CEH pigs, 6 out of 10 pigs developed lung lesions, which was not different from the CCH (OR = 2.7, *p* = 0.34) and EEH groups (OR = 3.5, *p* = 0.18).

#### 3.3.5 Lung bacteriology and histology

Lung bacteriology was assessed to evaluate the presence of *A. pleuropneumoniae* in lung tissue (Figure 10B). In 6 CCH pigs, 7 CEH pigs and 5 EEH pigs *A. pleuropneumoniae* was successfully re-isolated (out of 10 pigs per treatment). EEH pigs tended to have a lower bacteriological score after re-isolation of *A. pleuropneumoniae* compared with CCH and CEH pigs (housing effect, *p* = 0.05). In 4 out of 5 EEH pigs only low levels (score 1 out of 3) of *A. pleuropneumoniae* were re-isolated, whereas in CCH and CEH pigs most pigs had the highest bacteriological score (score 3 out of 3).

To further address the impact of co-infection on lung morphology, a histological evaluation of the lung samples was performed (Figures 10C–F). In the EEH pigs the patho-histological lung tissue score (overall histology score) was significantly lower compared with the CCH and CEH pigs (housing effect, *p* < 0.01), meaning that the extent of inflammation in lung tissue was lower in these pigs. The exudative component score was affected by housing (*p* < 0.01), with lower scores for EEH pigs as compared to CCH pigs, with CEH pigs being in between. The interstitial alveolitis score was lower for EEH pigs compared with CCH and CEH pigs (treatment effect, *p* = 0.01). Interstitial peribronchiolar and perivascular lympho-monocytic cell infiltration did not differ between housing treatments (*p* = 0.21).

## 4 Discussion

In this study the impact of enriched housing applied in different life phases of young pigs on development of immunity and disease outcome after infection was investigated. Pigs were housed in an enriched environment from birth (EEH), a conventional environment from birth (CCH), or switched from conventional to enriched housing at weaning (CEH). It was shown that EEH pigs had a differently developed immune response after weaning and displayed a lower disease severity in the used co-infection model starting with PRRSV followed by an *A. pleuropneumoniae* infection compared to CEH or CCH.

### 4.1 Impact of housing treatment on immune cells and function during the nursery period

The immune system after birth is not fully functional and is shaped in the following weeks and months under the influence of microbial colonization, environmental conditions and physiological regulators. Perinatal conditions, such as infections in early life, may have long-term effects on immune cell composition or state in different tissue compartments, the bone marrow or stem cell progeny (42), and thereby influence immune responses to challenges. In this experiment, even though WBC counts before weaning did not differ between enriched and conventionally housed pigs, the increase in white blood cells and specifically of granulocytes after weaning was stronger in EEH pigs than in CCH or CEH pigs. An increase of white blood cells and granulocytes after weaning is repeatedly reported and has been related to weaning stress, and this also holds for the decrease in NK cells that we found (43, 44). The recruitment of leucocytes in the transitional phase of weaning is often also interpreted as a response to hygiene conditions and pathogen pressure (5). Also changes in WBC counts and cytokine profiles after *in vitro* LPS stimulation were treatment dependent. EEH pigs showed a peak in WBC counts six days after weaning, after which counts decreased again, whereas in CCH and CEH pigs WBC counts more gradually increased over time. This indicates a faster response of WBC to weaning in EEH pigs than in CCH and CEH pigs. In line with this, IL1β concentrations six days after weaning and IL6 concentrations on day 20 after weaning were higher in EEH pigs than in CCH and CEH pigs in response to *in vitro* LPS stimulation, which indicates a faster or more adequate inflammatory response to LPS (45). In a previous study this effect of enrichment was already present before weaning, as IL1β and TNFα concentrations following an LPS challenge were higher in enriched housed pigs compared with conventionally housed pigs at two days before weaning (46). Thus, the increased pro-inflammatory reaction of EEH pigs after weaning in our study indicates a stronger innate immune response, which possibly enables those pigs to respond more adequately to infections in the nursery period.

A housing effect was also found in local alveolar immune cells of pigs at day 60, as CCH and CEH pigs tended to have a lower proportion of alveolar macrophages identified by expression of CD163+, CD172+ and CD172+CD163+ cells compared with EEH pigs. The lower proportion of alveolar macrophages in the CCH and CEH pigs may indicate that these pigs experienced a stronger inflammatory response in the lung (47). Hence, development of the lung immune system prior to co-infection seemed to differ between CCH and CEH pigs compared with EEH pigs. Interestingly, van Dixhoorn et al. (21) showed in an experimental setting that pigs from enriched housing had a lower proportion of TLR4+ and CD172a+/TLR4 macrophages than conventionally housed pigs. This lower percentage of macrophages with TLR4+ markers in enriched housed pigs was suggested to be related to a lower sensitivity of alveolar macrophages for LPS compared with the conventionally housed pigs. The current study was performed on a high health farm and the composition of lung cells was similar to that found by van Dixhoorn et al. (21). However, the percentage of CD14+TLR+ cells was higher in the EEH group than in the CEH or CCH group in the current study, whereas van Dixhoorn et al. (21) found opposite results. The contradicting results may be caused by the different microbial environment in animal experiment facilities and farm conditions, which impact the composition of broncho-alveolar cells and the activation of macrophages in the BALF. Therefore, it may be concluded that environmental conditions affect immune development of young pigs.

Given the effect of housing conditions on the response to weaning and the differences in lung macrophage expression prior to co-infection, enriched housed pigs in this study might have a different immune competence than conventionally housed pigs already before weaning. This is likely a result of exposure to a more complex and diverse environment due to pre-weaning socialization with another litter and manipulation of rooting materials in a larger environment (48, 49). This exposure may enhance the development of the pigs’ innate immune system and microbiota maturation and composition (50), thereby changing immune competence (46). In line with this, also others found long-term effects of environmental enrichment on immune (re)activity in pigs (22). More research to entangle the different effects of environmental enrichment and socialization prior to weaning on immune system development and on the response to weaning is required to increase knowledge of their consequences for health and welfare of pigs.

In the current study, CEH pigs differed in some immune variables six days after weaning compared with CCH and EEH pigs. CEH pigs had increased numbers of lymphocytes, less granulocyte counts and tended to have less NK cells than CCH and EEH pigs during six days after weaning. No to little differences between immune variables after weaning were expected in CCH and CEH pigs due to the same housing conditions before weaning, and the short time window between weaning and the first sample moment at six days after weaning. However, the differences may be related to the on-going adaptation of the CEH pigs and their immune system to the introduction of enrichment after weaning, on top of adaptation to the weaning process itself. Others investigating a change in housing from barren to enriched and vice versa, did find a pronounced effect of post-switch housing on immune (re)activity, as reflected in natural and specific antibody titers and relative percentages of several immune cells, and/or a complex interaction between pre-and post-switch housing over a 130-day study period (22). Based on these results, it was shown that immune development can be influenced by a change in housing conditions in the early life of a pig, but sufficient time for adaptation of the immune system is likely needed to benefit immune competence.

Precision-cut lung slices provide an *ex vivo* model system with an organotypic architecture of the lung, which can model local immune responses, mucus production and inflammatory responsiveness to lung infections (51). The system has been used to investigate infectious diseases and virus tropism in pigs in mono-and co-infection models (52, 53). In this study the PCLS model was used to investigate the possible impact of environmental enrichment on the local inflammatory response of the lung mucosa, in addition to the measured immunological responses. For this purpose, viral and bacterial mono-and co-infections with swine influenza A virus and *A. pleuropneumoniae* were used. Swine influenza A was used in the *ex vivo* co-infection model (instead of PRRSV) as infection with swine influenza A targets the lung epithelial cells whereas PRRSV infection only targets lung macrophages, and therefore this *ex vivo* model could give different information compared with the *in vivo* model. The *ex vivo* infections induced an expected inflammatory response, which is a characteristic for an influenza virus infection with increased mRNA levels of IFN-*β* and IL-8 after *A. pleuropneumoniae* infection. In addition, TLR4 was studied as it is a relevant receptor for LPS recognition and gram-negative bacteria. The observed reduction of TLR4 mRNA at 24 h after LPS stimulation was also seen after induction of acute lung injury in mice (54). Juarez et al. (55) have shown that LPS triggers mRNA expression shortly after exposure of LPS and that the mRNA expression in alveolar macrophages and bronchial epithelial cells reduced after 24 h. As we did not find differences in the studied mRNA expression patterns between the three environmental treatment groups, it may be hypothesized that environmental enrichment may have limited effect on the mucosal responsiveness in lung mucosal tissue. The response to viral and bacterial infection of the appropriate cytokines likely reflects the responsiveness of the tissue in the PCLS. Although the *ex vivo* PCLS do reflect the complexity of the cell structure of *in vivo* lung tissue, it only partly corresponds with the *in vivo* response to a co-infection. We can therefore conclude that *ex vivo* PCLS is at this moment not yet an ideal replacement for animal models. Nevertheless, we still continue to work on various alternative systems to reduce the number of animal studies, but *in vivo* generated results are still required to validate *ex vivo* results.

### 4.2 Response to co-infection after different housing treatments

To address the influence of environmental enrichment of the competence to counteract with infections a viral-bacterial co-infection model was used. The used *A. pleuropneumoniae* infection applied as mono-infection induces no or only mild clinical infections, however, in combination with PRRSV a more severe, but still moderate course of disease and a stronger pathology in the lungs can be induced. This enables to compare various background conditions to study infection susceptibility.

Apart from clear treatment effects on pre-infection immunity, also the response to the co-infection differed between treatments, showing a long term effect of the pigs’ housing conditions on their immune competence. EEH pigs had the most pronounced change in rectal temperature following both PRRSV and *A. pleuropneumoniae* infection, marked by fastest rise as well as fastest recovery in rectal temperature after the infections. This is in line with findings of van Dixhoorn et al. (21), who hypothesized that the faster drop in body temperature after *A. pleuropneumoniae* in life-long enriched housed pigs indicates a lower impact of this secondary infection, possibly due to a more adequate response to the primary PRRSV-infection compared with conventionally housed pigs.

EEH pigs showed a reduction in histological signs of disease in the lungs after the co-infection as interstitial alveolitis was lower in those pigs compared with CCH and CEH pigs. In addition, less EEH pigs tended to have lung lesions compared with CCH pigs, and less *A. pleuropneumoniae* tended to be re-isolated after co-infection from EEH pigs compared with CCH and CEH pigs. Also a faster decline in viral load following the primary PRRSV infection was seen in EEH pigs as compared to CCH and CEH pigs. Overall, the lower susceptibility to disease and faster viral clearance in EEH pigs to the lung challenge is comparable with the previous study of van Dixhoorn et al. (21) using the same pathogens and similar social and environmental enrichments. This previous study was carried out in an experimental setting whereas the current study took place on a commercial (high health) farm. Hence, with our study, we can conclude that also under commercial conditions environmental enrichment from birth onwards mitigates the impact of lung infection on pigs.

CEH pigs had a response to co-infection that was either similar to CCH pigs, or in between CCH and EEH pigs, with the exception of changes in rectal temperature and neutrophil percentage in response to infection, which differed from both other groups. The CEH treatment had an intermediate number of pigs with lung lesions and an intermediate score of local lung inflammation (exudative component). It confirmed our hypothesis that enrichment applied from weaning onwards would have an intermediate beneficial effect on disease susceptibility and thus lung damage. In CEH pigs, signs of interstitial alveolitis and amount of re-isolation of *A. pleuropneumoniae* were similar to those of CCH pigs, being higher than EEH pigs, which indicates that CEH pigs suffered more from the *A. pleuropneumoniae* infection than EEH pigs. Surprisingly, mean rectal temperature of CEH pigs in response to PRRSV was often lower than that of CCH and EEH pigs, but the mean rectal temperature after *A. pleuropneumoniae* was in between EEH and CCH pigs again. Thus, the switch from conventional housing prior to weaning to enriched housing after weaning seemed to slightly decrease disease susceptibility to co-infection with PRRSV and *A. pleuropneumoniae* compared with life-long conventionally housed pigs. Enrichment from birth seemed, however, to be most beneficial in decreasing disease susceptibility to this co-infection, possibly due to the importance of early life programming in shaping the immune system (56).

The current study showed that a switch in housing conditions at weaning led to minor changes in immune responsiveness shortly after weaning. However, on the long run, the switch in housing conditions did benefit the pigs’ health as the effect of the CEH treatment on disease severity after co-infection was intermediate compared with the CCH and EEH group. This indicates that the immune system of these CEH pigs has been developed in the enriched environment after weaning in such a way which decreased disease severity compared to CCH pigs. It is unknown whether and to what extent a switch to enriched housing at a later age would affect the immune competence of pigs.

## 5 Conclusion

This study shows that disease severity to a co-infection with PRRSV and *A. pleuropneumoniae* in pigs, manifested by more severe lung lesions, can be reduced by environmental and social enrichment applied from birth onwards under commercial conditions, possibly due to an enhanced immune competence. When provided with environmental enrichment from weaning onwards, the pigs showed a slightly reduced susceptibility to the lung infection, given the intermediate outcome of pathological measures in these pigs (between pigs housed conventional or enriched from birth onwards). These findings imply that enrichment applied directly after birth reduces disease severity after a challenge, and that effects when enrichment is applied later in life, such as around weaning, are less pronounced. More research is needed to elucidate the different mechanisms by which provision of environmental enrichment in different life stages affects immune competence in pigs and the subsequent consequences for their health and welfare.

## Data Availability

The raw data supporting the conclusions of this article will be made available by the authors, without undue reservation.
