# Supplementary material for: Environmental enrichment affects immunity and reduces disease severity in pigs after co-infection, with stronger effects when applied from birth than from weaning
Source: Front Vet Sci. 2024 Dec 9;11:1511209. doi: 10.3389/fvets.2024.1511209 (PMC11667117; doi:10.3389/fvets.2024.1511209)
Supplement: Supplementary file 1 [file Table_1.DOCX]

**Supplementary Table 1.** Blood phenotyping on three different days (D) in absolute numbers^1^ (± SD) [%] of pigs with different housing (H) treatments (CCH = conventional-conventional housing, CEH = conventional-enriched housing, EEH = enriched-enriched housing)

|  | **Day 26** | | | **Day 34** | | | **Day 56** | | | ***P*-values** | | | |
| --- | --- | --- | --- | --- | --- | --- | --- | --- | --- | --- | --- | --- | --- |
| **Type** | **CCH** | **CEH** | **EEH** | **CCH** | **CEH** | **EEH** | **CCH** | **CEH** | **EEH** | **H** | **D** | **H*D** |  |
| CD3+ | 4.84 (1.00) [35.0] | 4.57 (0.74) [39.7] | 5.41 (0.97) [35.9] | 10.4 (14.6) [35.0] | 6.11 (1.60) [40.0] | 6.42 (1.24) [32.4] | 5.92 (2.17) [30.8] | 5.37 (1.46) [34.6] | 5.72 (1.64) [33.0] | 0.94 | <0.01 | 0.92 |  |
| CD4+ | 2.78 (0.65) [19.8] | 2.44 (0.39) [21.3] | 2.71 (0.65) [18.2] | 5.93 (8.63) [19.7] | 3.30 (0.75) [21.9] | 3.50 (0.73) [17.7] | 2.76 (1.24) [14.5] | 2.31 (0.45) [15.1] | 2.68 (0.73) [15.5] | 0.68 | <0.01 | 0.88 |  |
| CD3+/CD8+ | 1.77 (0.46) [12.9] | 1.54 (0.36) [13.3] | 2.09 (0.44) [13.9] | 2.81 (3.40) [10.6] | 1.82 (0.53) [11.8] | 1.90 (0.30) [9.5] | 2.75 (1.85) [13.3] | 2.08 (0.67) [13.3] | 2.02 (0.50) [11.8] | 0.65 | 0.05 | 0.34 |  |
| CD3-/CD8+ | 2.4 (0.70)^b^ [18.2] | 2.01 (0.60)^b^ [17.3] | 3.30 (0.94)^a^ [21.8] | 2.00 (1.96) [8.3] | 1.22 (0.60) [7.6] | 1.31 (0.61) [6.5] | 1.07 (0.43) [6.2] | 0.91 (0.38) [5.8] | 0.97 (0.23) [5.7] | 0.04 | <0.01 | <0.01 |  |
| CD172+ | 4.87( 2.33) [36.8] | 3.55 (2.33) [31.2] | 4.31 (1.66) [28.5] | 6.28 (1.65)^b^ [37.8] | 5.21 (1.53)^b^ [34.3] | 10.5 (6.04)^a^ [44.9] | 6.57 (3.58) [39.3] | 5.75 (1.57) [37.0] | 6.66 (1.24) [39.0] | <0.01 | <0.01 | 0.05 |  |
| CD21+ | 1.9 (0.60) [14.0] | 1.86 (0.78) [16.0] | 2.57 (0.67) [16.9] | 4.12 (5.27) [15.1] | 2.24 (1.10) [14.3] | 2.34 (0.56) [11.7] | 2.5 (0.77) [14.3] | 2.14 (0.66) [14.0] | 2.35 (0.68) [13.5] | 0.21 | 0.08 | 0.07 |  |
| ^a,b^ Different superscript letters indicate a difference between values in one row on the same day (*P* < 0.05).  ^1^ Absolute numbers *10^6^/mL | | | | | | | | | | | | |  |
